# High‐frequency ultrasonography index in evaluation of pincer nail

**DOI:** 10.1111/srt.13306

**Published:** 2023-03-16

**Authors:** Qiaochu Zhou, Wei Wang, Jie Xu, ZhiWei Chen

**Affiliations:** ^1^ Wenzhou Hospital of Integrated Traditional Chinese and Western Medicine Affiliated Hospital of Integrated Traditional Chinese and Western Medicine of Shanghai University, Dermatology Department Wenzhou China

**Keywords:** high‐frequency ultrasonography, HF‐USG, nail, nail unit, pincer nail, pincer nail deformity

## Abstract

Background: The measurements of width index, height index, and curvature index were used for assessment of the curvature severity. Nevertheless, both sides of the nail root are buried subcutaneously, impossibility in measuring the width index correctly.

Materials and Methods: We developed a technique to measure the index under high‐frequency ultrasonography (HF‐USG).

Results: There was good agreement between the HF‐USG index and the result examined after surgery.

Conclusion: The observation on HF‐USG helps to distinguish between ingrown nail and pincer nail. The HF‐USG index will be useful in the examination and measurement of nail roots buried subcutaneously or nail penetration under the hypertrophic lateral nail fold, and comparing the effectiveness among treatments for pincer nail objectively.

1

Pincer nail is transverse overcurvature that pinches the nail bed. In the past, the measurements of width index, height index, and curvature index^1^ by visual observation were used for assessment of the curvature severity. However, both sides of the nail root that are buried subcutaneously caused imprecision in measuring of the pincer nail. Pincer nail deformity has three types, including the omega type (type1), the plicated nail (type2), and the tile‐shaped nail (type3). The plicated nail type is similar to the ingrown nail, which causes the nail edges to descend into the lateral nail fold and hypertrophy of the lateral and distal nail fold. In some situations, hypertrophy of the lateral and distal nail fold, and the excess of soft tissues that cover the plate interfered with the measurement. The correct measurement of the pincer nail structure covered with hypertrophic nail fold is impossible. The evaluation system of curvature index has some drawbacks including when both nail sides are buried in the lateral nail fold. Consequently, it is important to develop a more effective evaluation system.

The evaluation of the pincer nail should be the evaluation of the complete structure. The past width index leads to a decrease in sensitivity and inaccurate results due to the neglect of the width of both sides of the nail root that are buried subcutaneously, especially when the degree of overcurvature is not serious. The past curvature index is based on the evaluation of the bending degree of the distal nail plate, but once it is covered by soft tissues, it will also cause detection interference or even failure. Our concept is to combine high‐frequency ultrasonography (HF‐USG) with visual observation to overcome covered tissues and obtain more objective indexes.^2^

To address this issue, we developed a new technique to measure the HF‐USG index under HF‐USG (Figure 1). The HF‐USG index was measured with Ultrasound SkinScanner‐DUB (Tpm taberna pro medicum) using a high‐frequency 22‐MHz probe. In order to facilitate the operation and avoid contamination, the probe was covered by the medical‐surgical film (ZHENDE Medical) when measuring. The apparent width of the nail plate between both sides of the lateral nail fold on the proximal was measured using a caliper as A1, and the width of both sides of the nail root that were buried subcutaneously under HF‐USG as A2‐3. When hypertrophy of the lateral and distal nail fold covered the plate, the apparent length of the nail tip between both sides of the lateral nail fold was defined as HF‐USG curvature index D1, the apparent width of the nail tip as E1, and the structure covered with hypertrophic lateral nail fold were defined as D2‐3 and E2‐3. The HF‐USG width index was defined as B/(A1+A2+A3). The HF‐USG curvature index was defined as (D1+D2+D3) /(E1‐E2‐E3). When the degree of transverse overcurvature is not severe, E2 or E3 may be added to E1. The addition or subtraction of the curvature index E needs to be determined by observing the shape of the nail with the instrument. In some cases, only one side is bent, or the overcurvature degree does not form a reverse fold. The measurements of A2‐3, D2‐3, and E2‐3 were obtained under HF‐USG. We modified the width index and curvature index as HF‐USG width index and HF‐USG curvature index; other index and calculations were the same as the original methods. Here, we first use HF‐USG to evaluate the pincer nail.

**FIGURE 1:**
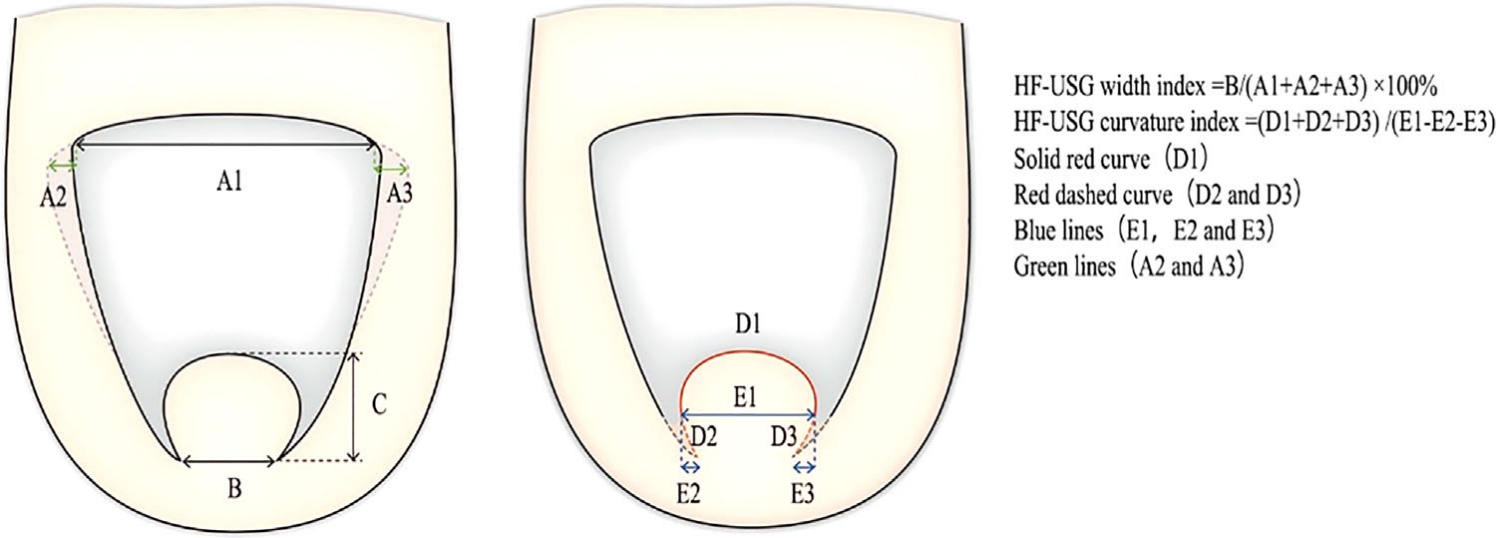
Illustration of this technique. The apparent width of the proximal nail plate defined was marked as A1 and the width of the nail root that was buried subcutaneously under high‐frequency ultrasonography (HF‐USG) as A2‐3 (green lines). When the hypertrophy of the lateral and distal nail folds covered the plate, the apparent length of the nail tip was defined as the HF‐USG curvature index D1, the apparent width of the nail tip as E1, and the structure covered with hypertrophic nail folds as D2‐3 and E2‐3 (red dashed curve and blue lines). Of note, the use of HF‐USG also required the observation of the transverse overcurvature direction of the distal nail plate, and the curvature direction is used to determine whether E2 and E1 are additive or subtractive, and likewise for E3.

A 34‐year‐old woman complained of the presence of an omega‐type pincer nail with an ache over the last 2 years. Physical examinations revealed an increase in the transverse curvature from the proximal to the distal nail of the right great toe. In addition, hypertrophy of the lateral and distal nail folds and both sides of the distal nail tip was not exposed and thus not observed (Figure 2A). The tips of the nail were buried subcutaneously, which caused inaccurate measurements of the pincer nail with the evaluation system of width, height, and curvature indexes. The width of the distal nail tip needs to be measured whether the evaluation system of the width index or the curvature index is used. However, this measurement method only reflects the apparent width of the distal nail plate. Therefore, we used HF‐USG to measure the width of the covered nail plate (Figure 2B‐C).

Next, the length of the distal nail plate was measured with the evaluation system of the curvature index, which showed that the distal nail plate was 10 mm (Figure 2D). The same issue also existed, that is, this measurement method only reflects the apparent width of the distal nail plate, whereas the length of the nail tip between both sides needed to be measured. When the surgery of correction of pincer nail deformity with a dermal flap was used for treatment, the removed nail plate was measured for the length of the distal nail tip, and the result was 14 mm, which was obviously not consistent with the preoperative data of 10 mm (Figure 2E). Preoperatively, HF‐USG was utilized to measure the length of the covered nail tip D2 and D3, and the apparent length of the distal nail plate plus D2 and D3 were 14 mm, which was consistent with the postoperative data (Figure 2F).

In this case, HF‐USG was mainly used to complement the data of the covered nail tip, which was combined with the data obtained by conventional measurements of the apparent nail plate for an accurate preoperative measurement. Importantly, HF‐USG should also measure the transverse overcurvature direction of the distal nail plate, and the curvature direction is used to determine whether E2 and E1 are additive or subtractive, and likewise for E3.

A 70‐year‐old woman presented to the hospital with a painful omega‐type pincer nail for the last 11 years. Physical examinations showed an increase in the transverse curvature from the proximal to the distal nail of the left great toe (Figure 3A). The completely exposed distal nail plate can be measured with the conventional method to obtain height and curvature indexes. However, if the measurement of the width index is needed, the width index data of the proximal nail root which is covered by HF‐USG should be completed. Nonetheless, when the width index is needed to be measured, HF‐USG should be used to complement the data of the width index of the proximal nail root that is covered. The apparent width A1 of the proximal nail plate defined was 12.4 mm. The width A2 of the nail root buried subcutaneously under HF‐USG (red line) was 1.8 mm (Figure 3B), and A3 was 1.9 mm. In this case, we treated with zigzag nail bed flap^3^ and had no recurrence at 6 months follow‐up. The width of the removed nail plate during surgery was 16.8 mm. There was favorable consistency between the HF‐USG index and the result of the postoperative examination.

**FIGURE 2:**
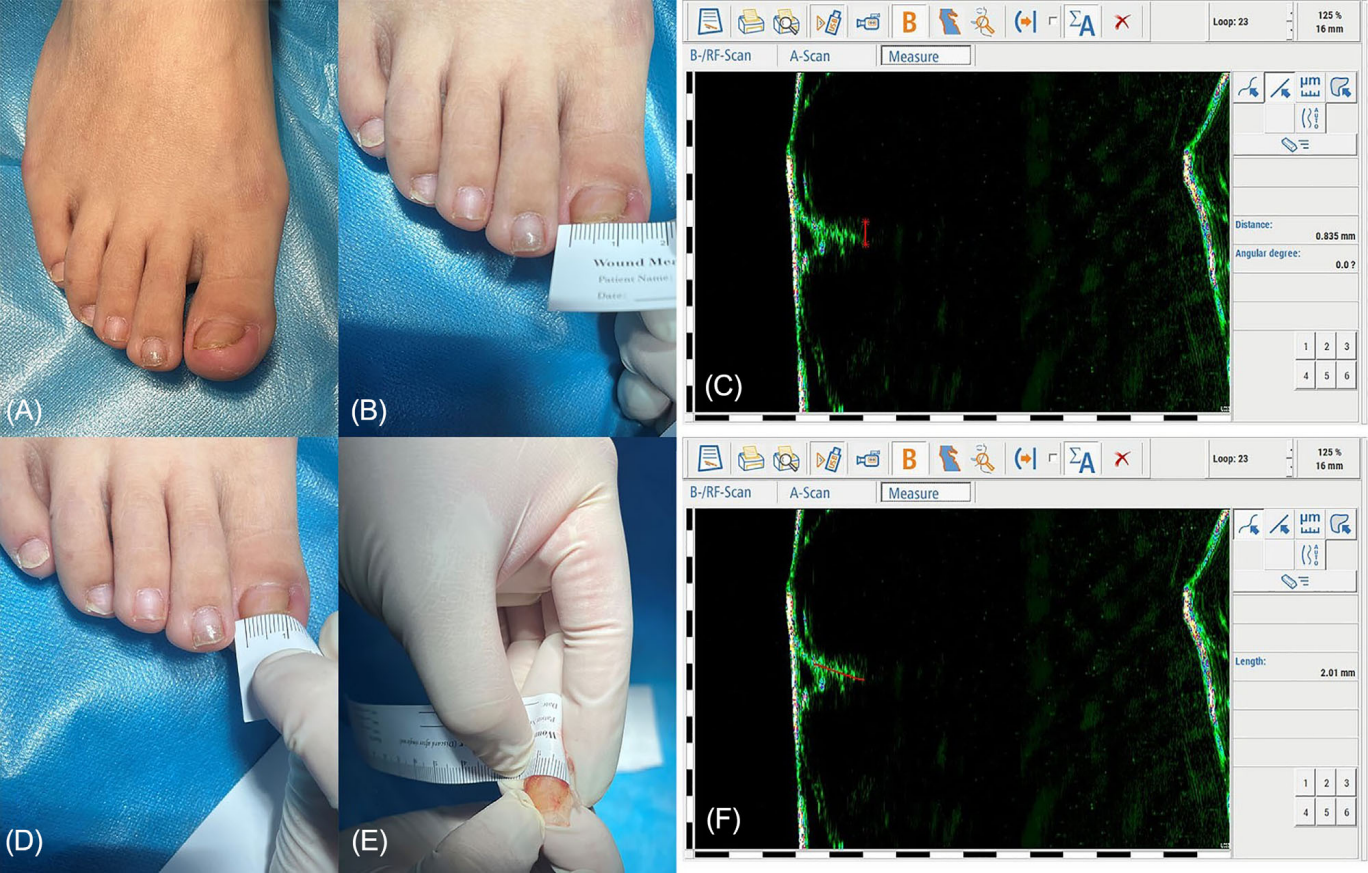
The use of high‐frequency ultrasonography (HF‐USG) complemented the data of the covered nail tip, which were combined with the data obtained from the conventional measurement of the apparent nail plate for an accurate preoperative measurement. (A) The case of the pincer nail with unexposed hypertrophy of the lateral and distal nail folds and both sides of the distal nail tip. (B) The apparent width of the distal nail plate. (C) The width E2 of the covered nail plate complemented by HF‐USG. (D) The length (10 mm) of the distal nail plate measured with the evaluation system of the curvature index. (E) The length (14 mm) of distal the nail tip of the nail plate removed during surgery, clearly inconsistent with the preoperative data of 10 mm. (F) Preoperatively, the length D2 of the nail tip of the covered part complemented using HF‐USG, and D3 was also measured, which were added with D1 data, consistent with the postoperative data.

**FIGURE 3:**
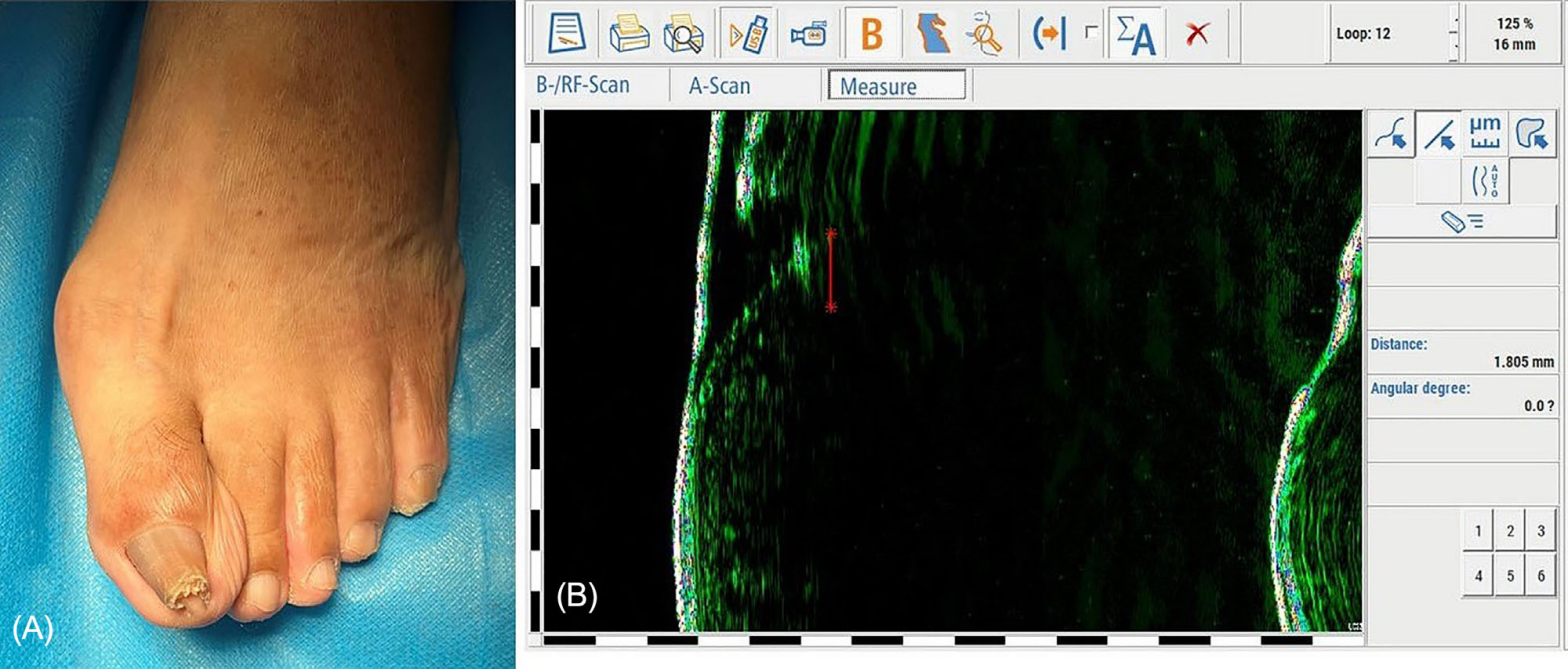
The completely exposed distal nail plate can be measured with the conventional method to obtain height and curvature indexes. The use of high‐frequency ultrasonography (HF‐USG) complemented the data of the covered nail root, which were combined with the apparent width of the nail plate to obtain a more accurate width index. (A) The case of the omega‐type pincer nail. (B) A2 of the HF‐USG width index (1.8 mm) and the width of the nail root buried subcutaneously under HF‐USG (red line).

**FIGURE 4:**
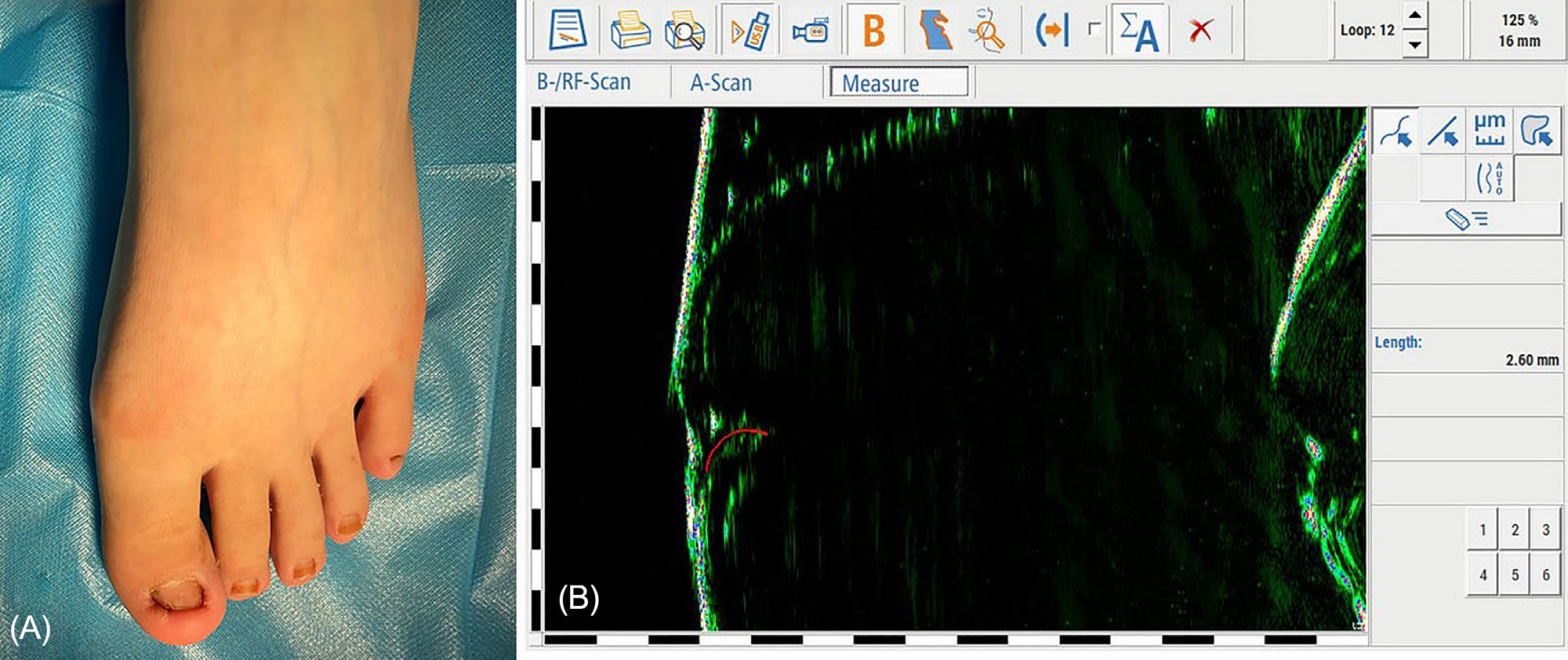
The use of high‐frequency ultrasonography (HF‐USG) overcomes the interference of the lateral nail groove and produced granulation tissues. (A) In the case of the plicated nail type of pincer nail with hypertrophic lateral walls similar to the ingrown nail. (B) D2 of the HF‐USG curvature index (2.6 mm) the length of the nail tip covered with hypertrophy of the lateral and distal nail folds under HF‐USG (red curve).

In this case, HF‐USG was used to complement the data of the covered nail root, which was combined with the apparent width of the nail plate to obtain a more accurate width index. Especially, with the width index plus A1 and A2 data obtained by HF‐USG was more sensitive for the measurement of early transverse overcurvature, facilitating the early detection and intervention of pincer nails. For severe transverse overcurvature, the combination of the width index plus A1 and A2 data obtained by HF‐USG with curvature and height indexes can reflect the change of the whole nail plate from proximal to distal in a more three‐dimensional way.

A 25‐year‐old woman presented to the hospital due to 7 years of the plicated nail type of pincer nail with hypertrophic lateral walls similar to the ingrown nail. Physical examinations indicated that lateral edges were sharply bent to form vertical sheets, which pressed into the lateral nail groove, thus producing granulation tissues of the left great toe (Figure 4A). The distal nail tip was not exposed and was not observed.

The evaluation system of the height index is difficult to be used for measurement in the case of severe covering. Therefore, the evaluation system of the curvature index was used. However, the lateral nail groove and produced granulation tissues also interfere with measurements with the evaluation system of the curvature index, which was overcome by HF‐USG in this case. The length D2 of the nail tip covered with hypertrophy of the lateral and distal nail folds under HF‐USG (red curve) was 2.6 mm (Figure 4B). The apparent length D1 of the nail tip between both sides of the lateral nail folds was 10.5 mm, and D3 of the HF‐USG curvature index was 2.8 mm. In this case, we treated with correction of pincer nail deformity with dermal flap^4^ and had no recurrence at 5 months follow up. The traced length of the removed nail plate removed during surgery was 15.4 mm. Excellent agreement existed between the HF‐USG index and the result of the postoperative examination.

In this case, the use of HF‐USG mainly overcomes the interference of the lateral nail groove and produced granulation tissues, and seems to reflect the potential of measuring ingrowing nails with hypertrophic lateral walls.

The HF‐USG index was useful in the examination of the pincer nail structure under the lateral nail fold. The study for measuring the ultrasound structure of the nail plate showed that the sound speed could be changed, and the ultrasound velocity in the nail plate is higher than in soft tissue.^5^ We could choose the HF‐USG index required according to the specific situation. We modified the measurement method through HF‐USG, combined with values measured by visual observation, which can exactly mirror the structure of the pincer nail and yield accurate measurement values.

Our measurement technology can match the two original measurement methods, which is more accurate in measurement and solves the issue of measuring the pincer nail structure covered with hypertrophic lateral nail fold. Specifically, this technology compensates for the fact that the two original measurement methods are limited by visual observation, that is, observation and measurement can be completed by HF‐USG even if the pincer nail structure is covered. Therefore, the treatment effect can be evaluated objectively with this technology.

The result can be more accurate and closer to the real situation by adding the width of the subcutaneous part on both sides of the nail root to the calculation, especially when the transverse overcurvature is not severe. For early transverse overcurvature, neglect can occur during the calculation of the width index with the original method, which is not conducive to early detection and intervention of the pincer nail. As a result, it is highly recommended to calculate the width index with our HF‐USG index.

To our knowledge, this is the first study of HF‐USG on the pincer nail. We believe that our technology is valuable for differential diagnosis, severity assessment, treatment guidance, and efficacy evaluation of both ingrown toenails and pincer nails, as well as trapezoidal nail. A small sample size is considered a limitation. The technique needs further verification with a large sample size and summary, including correlating with a healthy control group. Therefore, we will conduct more in‐depth studies to establish novel assessment systems.

## CONFLICT OF INTEREST STATEMENT

The authors declare that there is no conflict of interest that could be perceived as prejudicing the impartiality of the research reported.

## Data Availability

The datasets used and/or analyzed during the current study are available from the corresponding author upon reasonable request.
